# Determination of Ferulic Acid in *Angelica sinensis* by Temperature-Controlled Hydrophobic Ionic Liquids-Based Ultrasound/Heating-Assisted Extraction Coupled with High Performance Liquid Chromatography

**DOI:** 10.3390/molecules25153356

**Published:** 2020-07-24

**Authors:** Hongwei Wu, Qianqian Huang, Shujun Chao, Jie Yu, Shengrui Xu, Feng Wang, Xuefang Shang, Yan Zhu

**Affiliations:** 1 School of Basic Medical Sciences, Xinxiang Medical University, Xinxiang 453003, China; wujihuaxue2017@sina.com (Q.H.); chaoshujun1979@163.com (S.C.); yujie_927@163.com (J.Y.); wfeng100@126.com (F.W.); xuefangshang@126.com (X.S.); 2 School of Chemistry and Chemical Engineering, Henan Normal University, Xinxiang 453003, China; xushengrui@126.com; 3 Department of Chemistry, Zhejiang University, Hangzhou 310028, China

**Keywords:** ferulic acid (FA), temperature-controlled hydrophobic ionic liquids-based ultrasound/heating-assisted extraction (TC-ILs-UHAE), high-performance liquid chromatography (HPLC), *Angelica sinensis*

## Abstract

Hydrophilic ionic liquids are often used to extract the active ingredients of medicinal plants, while hydrophobic ionic liquids are rarely used to directly extract solid samples. In this paper, a simple, novel and efficient temperature-controlled hydrophobic ionic liquids-based ultrasound/heating-assisted extraction (TC-ILs-UHAE) procedure coupled with high-performance liquid chromatography (HPLC) was developed and applied to the determination of ferulic acid (FA) in Chinese herbal medicine *Angelica sinensis*. During the extraction procedure, hydrophobic ionic liquids (ILs) were dispersed into water to form cloudy solution (fine droplets) with the aid of ultrasound and heating simultaneous. After extraction, phase separation was easily achieved by centrifuging at 0 °C. Among all ILs used, 1-butyl-3-methylimidazolium bis(trifluoromethanesulphonyl)imide ([C_4_mim]NTf_2_) exhibited the highest extraction ability and the possible extraction mechanism was discussed. Additionally, the synergistic effect of heating and ultrasound on the extraction efficiency was investigated. Under the optimized conditions, a good linearity was observed with correlation coefficient (*r*) of 0.9995. The limit of detection of FA (LOD, *S*/*N* = 3) was 9.6 µg/L and the spiked recoveries of FA for real samples were in the range of 91.67 to 102.00% with relative standard deviation (RSD) lower than 3.87%. Compared with the traditional extraction methods, the proposed method gave the highest yield of FA and had the shortest extraction time. Therefore, this method is a potential simple, green and highly efficient technique and expected to be applied to the extraction of other bioactive ingredients in medicinal plants.

## 1. Introduction

*Angelica sinensis (A. sinensis)* is the rhizome of *Angelica sinensis (Oliv.) Diels* (Umbelliferae family). It has been widely used not only as a health food and drug in Asia but also as a dietary supplement in women’s care in Europe [1]. Nearly 20% of the traditional Chinese medicinal preparations included in the Chinese Pharmacopeia contained *A. sinensis.* Based on the recent pharmacological studies, *A. sinensis* possesses the function of tonifying, lubricating the intestines, protecting the heart, enhancing immune function, anti-arrhythmic, anti-atherosclerotic, treating female irregular menstruation and preventing myocardial infarction events by inhibiting smooth muscle inflammation and platelet aggregation [2,3,4]. Many bioactive constituents including polysaccharides, phenolic acids and angelica lactone have been isolated from *A. sinensis* [5]. Among them, ferulic acid (FA) is an important and valuable phenolic acid whose content is used for assessing the quality of *A. sinensis* according to the Pharmacopoeia of China [6]. FA has many pharmaceutical properties such as antioxidant, anti-inflammatory and anti-tumor effects [7]. Therefore, it is very important to develop a simple, green, sensitive and reliable method for the extraction and determination of FA to control the quality of *A. sinensis*.

Until now, many pretreatment methods have been used for the extraction of bioactive components including FA from *A. sinensis*. The conventional approaches commonly used were heated reflux extraction (HRE) [8] and Soxhlet extraction [9]. These methods suffer from complicated operation, time-consuming and the use of a large amount of organic solvents. Therefore, instrumental assisted extraction methods of FA such as ultrasonic-assisted extraction (UAE) [10], high hydrostatic pressure extraction (HHPE) [11], supercritical fluid extraction [12] and pressurized liquid extraction (PLE) [2] were attracting more and more attentions due to their advantages of easy operation, time and solvent saving. Over the past few years, UAE has become increasingly popular and proven to be very practical owing to its low cost, easy operation and high extraction efficiency characteristics [13,14,15]. The ultrasonic would induce cavitation and shockwaves, leading to increasing solid–liquid extraction yield [16]. Thus, the plant tissues could be easily disrupted, leading to quick penetration of extraction solvents into the plant tissues and extraction of the chemical components. However, the use of toxic, volatile, flammable organic solvents, like acetonitrile, methanol and ethanol, are still highly problematic for the lab workers and environment. Therefore, seeking for the safe and environmentally benign extraction solvents and processes is increasingly important in the development of sample pretreatment techniques.

Recently, ionic liquids (ILs) have become promising alternatives to the traditional organic solvents employed in sample preparation because of their unique properties, such as negligible vapor pressure, high thermal stability, non-flammability and good solubility for many inorganic and organic compounds [17]. Therefore, ILs have been extensively applied as substituents for conventional organic solvents in various extraction processes including salt-induced liquid–liquid extraction (SI-LLE) [18], dispersive liquid-phase microextraction (DLPME) [19], microwave-assisted extraction (MAE) [20], pressurized liquid extraction [21], ultrasonic-assisted extraction [22,23,24] and temperature-controlled liquid phase micro-extraction (TC-LPME) [25,26,27,28]. In this context, hydrophobic ILs are usually used to extract liquid samples, while hydrophilic ILs are mostly used to extract solid samples. Due to the high viscosity of hydrophobic ILs and the difficulty of phase separation, it is rarely reported that hydrophobic ILs were directly used to extract solid samples [29]. In most cases, water and organic solvent were firstly used to extract the trace components in solid samples, and then hydrophobic ILs were selected to extract and concentrate the trace components in the extracts [25]. This process was not only complicated and time-consuming, but also easy to cause measurement errors. Recently, TC-LPME has attracted more and more attention. Hydrophobic ILs were used as the extraction solvents and dispersed completely into the aqueous phase under the drive of temperature in this method. Compared with conventional dispersed liquid phase micro-extraction, this method is efficient and environmentally friendly. However, this method has rarely been applied directly to the extraction of solid samples and there is still a lack of systematic research on the extraction mechanism. To our best knowledge, there is no report about temperature-controlled hydrophobic ILs based ultrasound/heating-assisted extraction (TC-ILs-UHAE) of FA from *A. sinensis*.

The present work aimed to establish an eco-friendly, easy operation and effective TC-IL-UHAE procedure coupled with high-performance liquid chromatography (HPLC) for the determination of FA in Chinese herbal medicine *A. sinensis*. Water, methanol and ILs with different anions and cations were selected as extraction solvents and their extraction efficiency were investigated. The possible extraction mechanism was also discussed. Moreover, the synergistic effect of heating and ultrasound on the extraction efficiency was investigated and parameters related to extraction and determination were optimized systematically. Furthermore, the extraction performance of the proposed method was compared with heating reflux extraction and Chinese Pharmacopoeia method for the extraction of FA in real samples.

## 2. Materials and Methods

### 2.1. Materials

**Figure 1:**
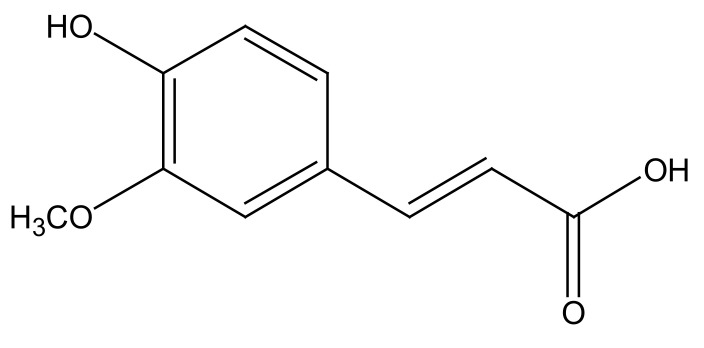
Chemical structure of ferulic acid (FA).

Ferulic acid (FA, ≥99.8%) was obtained from Aladdin Industrial Inc. (Shanghai, China). The chemical structure of FA is shown in Figure 1. All the ILs (99%), including 1-butyl-3-methylimidazolium hexafluorophosphate ([C_4_mim]PF_6_), 1-hexyl-3-methylimidazolium hexafluorophosphate ([C_6_mim]PF_6_), 1-octyl-3-methylimidazolium hexafluorophosphate ([C_8_mim]PF_6_), 1-butyl-3-methylimidazolium bis(trifluoromethanesulphonyl)imide ([C_4_mim]NTf_2_), 1-hexyl-3-methylimidazolium bis(trifluoromethanesulphonyl)imide ([C_6_mim]NTf_2_) and 1-octyl-3-methylimidazolium bis(trifluoromethanesulphonyl)imide ([C_8_mim]NTf_2_), were purchased from The Centre of Green Chemistry and Catalysis, LICP (Lanzhou, China) and used as received. Six batches of the rhizome of *A. sinensis* samples (NO. 1–6) from different cultivation regions were purchased from local drug store (Xinxiang, China). Sample NO. 1–4 are cultivated in Gansu province and sample NO. 5–6 are cultivated in Yunnan province. HPLC-grade methanol was provided by Tedia Company (Fairfield, OH, USA). Ultrapure water produced by Milli-Q academic water-purification system (Molsheim, France) was used throughout the experiment. All other chemicals were at least of analytical reagent grade.

The standard stock solution (100 mg/L) of FA was prepared in methanol and diluted with ultrapure water to obtain appropriate concentrations of working solutions. All the standard solutions were stored at 4 °C in the refrigerator and protected from light.

### 2.2. Equipment

HPLC analysis was performed with a Waters e2695 liquid chromatographic system (Waters, Milford, MA. USA) including a quaternary pump, a 2998 photo-diode array detector (PDA), an automatic sampler and a thermostatic column compartment. A Waters Symmetry C18 column (4.6 mm × 250 mm, particle size 5 μm) was used for the separation of FA extracted in the IL phase. The KQ-500V ultrasonic generator with an electrical power of 500 W was purchased from Kunshan Ultrasonic Instrument Co. Ltd. (Kunshan, China). The centrifugation was carried out on a high-speed freezing Heraeus Multifuge X1R centrifuge from Thermo Scientific (Waltham, MA, USA).

### 2.3. Sample Preparation

All samples were dried in a vacuum oven (Model 1400E, VWR Scientific Products, West Chester, PA, USA) to constant weight at 40 °C, then triturated with a pulverizer and passed through a 40-mesh sieve. After being homogenized, the dried powders of samples were collected and stored in a moisture controlled cabinet.

### 2.4. Temperature-Controlled Hydrophobic IL Based Ultrasound/Heating-Assisted Extraction

**Figure 2:**
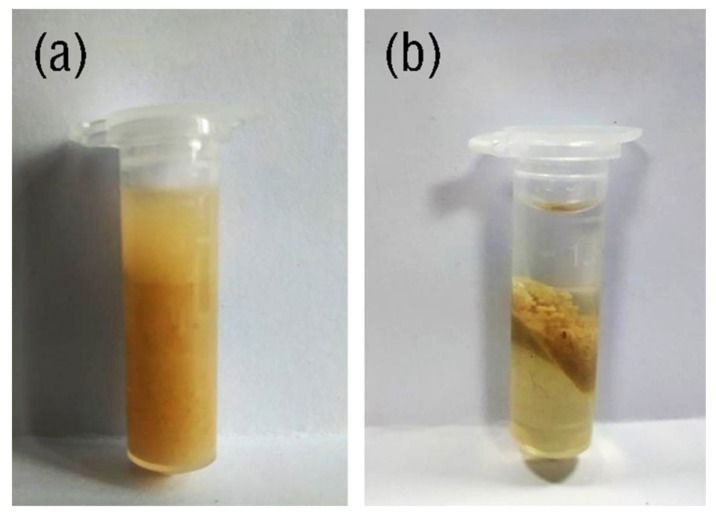
Scheme of centrifugal process, (**a**) before centrifugation and (**b**) after centrifugation.

**Figure 3:**
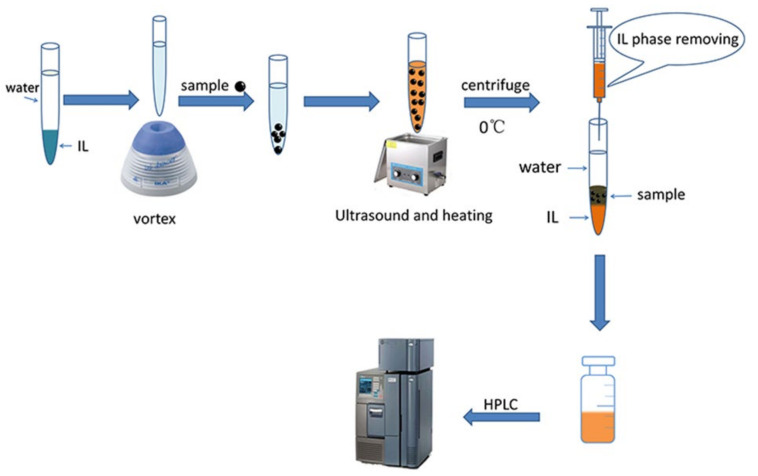
Schematic diagram of temperature-controlled hydrophobic ionic liquids-based ultrasound/heating-assisted extraction (TC-ILs-UHAE) method.

Hydrophobic IL, [C_4_mim]NTf_2_ (0.80 mL) and ultrapure water (1.20 mL) were added in a calibrated centrifuge tube and mixed at 60 °C in advance. The IL was dissolved completely and mixed with the water entirely under the force of ultrasound and heating. Subsequently, precisely weighed sample powder (50 mg) was added in the centrifuge tube and ultrasonic extracted for 9 min at 60 °C. The resultant mixture was centrifuged at a speed of 11,000 r/min for 5 min at 0 °C. After that, it can be seen from Figure 2 that a sandwich-like structure was formed. The top, middle and bottom phases were water, solid sample and IL phases, respectively. The IL phase was withdrawn using a syringe and filtered through 0.45 µm membrane filter. The clarified filtrate was diluted 5 times by mobile phase before HPLC analysis. A schematic diagram of TC-ILs-UHAE is shown in Figure 3.

The extraction yield of target analyte was determined as follows:

(1) $$\mathrm{yield}\;(\mathrm{mg}/g)=\frac{\mathrm{mean}\;\mathrm{mass}\;\mathrm{of}\;\mathrm{target}\;\mathrm{analyte}\;\mathrm{in}\;\mathrm{herb}\;\mathrm{samples}\;(\mathrm{mg})}{\mathrm{mean}\;\mathrm{mass}\;\mathrm{of}\;\mathrm{the}\;\mathrm{herb}\;\mathrm{samples}\;(g)}$$

The mean mass of target analyte in herb samples was calculated for 3 subsequent sample determinations under the optimized conditions. The mean mass of the herb samples was the average mass of three samples before extraction.

### 2.5. Conventional Reference Extraction Procedures

HRE and Chinese Pharmacopoeia methods were both selected as the references for extraction of FA from *A. sinensis* samples. Preliminary experiments shown that the optimal extraction conditions of the two procedures are as follows:

In HRE procedure, 200 mg of dried sample powder was added into the mixture of 8.00 mL [C_4_mim] NTf_2_ and 8.00 mL ultrapure water in a distillation flask. The sample was then extracted for 60 min under reflux at 80 °C using water bath with magnetic stirring. After that, the sample mixture was transferred into a 15 mL calibrated centrifuge tube and centrifuged at a speed of 11,000 r/min for 5 min at 0 °C. Subsequently, the IL phase was withdrawn using a syringe and filtered through 0.45 µm membrane filter. The clarified filtrate was diluted 5 times by mobile phase before HPLC analysis.

In Chinese Pharmacopoeia method, sample powder (200 mg) and 70% methanol (20 mL) were placed in a distillation flask. The distillation flask was weighed accurately. Then, hot reflux extraction was performed for 30 min. After cooled to the room temperature, the distillation flask was weighed again, and the lost weight was made up with 70% methanol. Subsequently, the sample mixture was shaken and filtered. The obtained filtrate was filtered through a 0.45 µm membrane filter before HPLC analysis.

### 2.6. Scanning Electron Microscopy Analysis (SEM)

Scanning electron microscopy (SEM) imaging was used to characterize the surface morphology of *A. sinensis* sample 1 before and after extraction. A Phenom XL Scanning Electron Microscope (Thermo Scientific, Waltham, MA, USA) in the secondary electron mode with 15 kV accelerating voltage was used to observe the microstructures of the raw and extracted samples. Before proceeding with imaging procedures, the samples were coated with a thin layer of platinum at room temperature.

### 2.7. HPLC Analysis

**Figure 4:**
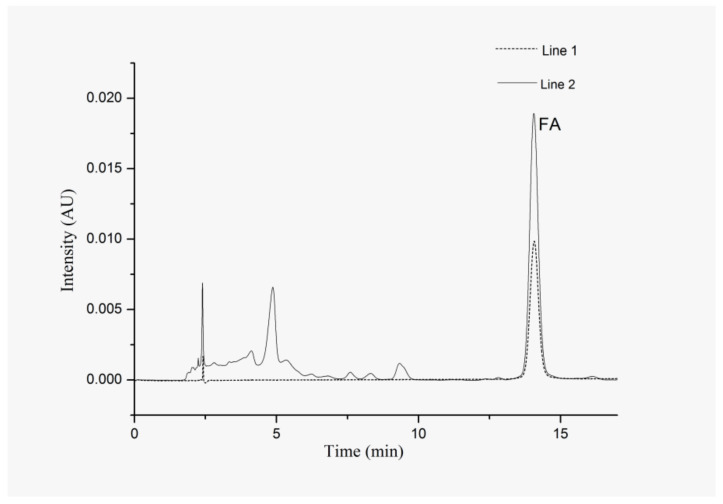
High-performance liquid chromatography (HPLC) chromatogram of 5 µg/mL FA standard solution (line 1) and the extract of sample 1 (line 2).

The diluted extracts were directly injected into the HPLC system. The mobile phase used was a binary mixture of methanol-0.2% phosphoric acid aqueous solution (32:68, v/v) at a flow rate of 1 mL/min. The injection volume was 10 μL and the column temperature was set at 35 °C. Peak areas of the samples were monitored at 323 nm. Triplicate injections were executed for the analysis of each sample. Chromatograms of FA obtained from standard solution and sample 1 extract are shown in Figure 4.

## 3. Results and Discussion

### 3.1. Optimization of TC-ILs-UHAE

In order to obtain high extraction yields, some important parameters influencing the extraction efficiency of FA were carefully explored by taking sample 1 as a representative. All single factor experiments were performed in triplicate and each datum presented in this work is expressed as mean ± standard deviation (SD).

#### 3.1.1. Selection of the Types of Extraction Solvents

**Figure 5:**
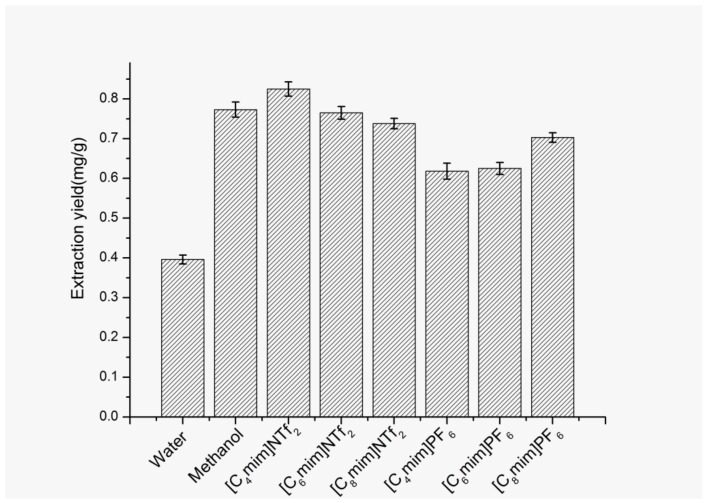
Effect of different extraction solvents on extraction yield of FA. Sample amount, 50 mg; volume of IL, 0.8 mL; extraction temperature, 60 °C; solid–liquid ratio, 1:40 (g/mL); ultrasonic time, 9 min; centrifugal speed, 11,000 rpm; centrifugal time, 5 min.

The types of ILs have a significant influence on the extraction efficiency. Yu [30] has reported the extraction of ferulic acid and caffeic acid from aqueous solution by using [C_4_mim]PF_6_ and [C_6_mim]PF_6_. In our proposed method, ILs with higher hydrophobicity and lower viscosity is expected to be beneficial to improve the extraction efficiency of FA in the solid herbal samples. Therefore, six kinds of ILs, [C_4_mim]PF_6_, [C_6_mim]PF_6_, [C_8_mim]PF_6_], [C_4_mim]NTf_2_, [C_6_mim]NTf_2_ and [C_8_mim]NTf_2_, were selected to extract FA. This selection enables us to systematically study the influence of the nature of the IL anion and cation on the extraction efficiency of FA. Moreover, pure water and methanol were also selected as the reference extraction solvents for evaluating the extraction efficiency of FA with different solvents. The results shown in Figure 5 indicate that the extraction ability of different extractants follows the order: [C_4_mim]NTf_2_ > [C_6_mim]NTf_2_ ≈ methanol > [C_8_mim]NTf_2_ > [C_8_mim]PF_6_ > [C_6_mim]PF_6_ ≈ [C_4_mim]PF_6_ > pure water. As can be seen from Figure 5, four interesting phenomena can be obtained: (I) The addition of ILs obviously improves the extraction yield of FA from *A. sinensis* compared with pure water as solvent. This could be ascribed to the multiple interactions, including hydrophobicity, π–π stacking interaction, dispersion force and hydrogen bonding between FA and imidazolium based ILs [30,31]. (II) For the ILs with the same cation but different anions, the extraction ability of [C_n_mim]NTf_2_ was higher than that of [C_n_mim] PF_6_ (*n* = 4, 6, 8). It is consistent with the order of the anion’s hydrophobicity (NTf_2_^−^ > PF_6_^−^). This suggests that the hydrophobicity of ILs is one important driving force underlying the extraction process. In addition, ILs with strong hydrophobicity more easily reach phase separation and reduce the solubility in the aqueous phase during centrifugation, which is also beneficial to increase the extraction efficiency. Moreover, the viscosity of extractant has a great influence on extraction efficiency. Low viscosity is not only conducive to the mass transfer rate, but also conducive to better dispersion of IL into the aqueous phase in the process of ultrasound and heating. The viscosities of NTf_2_^−^ based ILs are much lower than those of PF_6_^−^ based ILs [32]. This suggests that viscosity is also a key parameter affecting the extraction efficiency in the proposed method. (III) For the ILs with the same anions but different cations, the hydrophobicity of the ILs increases with prolonging the alkyl chain length of the IL cation. However, as shown in Figure 5, the increase in the alkyl chain length of the IL cation results in the decrease in the extraction ability ([C_4_mim]NTf_2_ > [C_6_mim]NTf_2_ > [C_8_mim]NTf_2_), which is opposite to the order of the cation’s hydrophobicity. To explain this phenomenon, steric hindrance effect between ILs and FA should be considered. The increase of alkyl chain of ILs leads to the increase of the steric hindrance effect. There are dispersion forces and hydrogen bond forces between ILs and FA, and the increase of steric resistance will reduce these two forces, lowering the extraction ability of ILs. That is to say, there is a competition between hydrophobicity and steric hindrance. Moreover, the viscosity of short chain ILs is relatively low, which is also beneficial to improve the extraction rate of FA. Based on the above factors, [C_4_mim]NTf_2_ has the highest extraction efficiency of FA. Another thing which should be noted is that for PF_6_^-^- based ILs, the extraction efficiency of FA follows the order: [C_8_mim]PF_6_ > [C_6_mim]PF_6_ ≈ [C_4_mim]PF_6_. This indicates that there is a delicate balance among all interactions between FA and ILs. (IV) It is noteworthy that the extraction ability of methanol is only similar to that of [C_6_mim]NTf_2_ and less than that of [C_4_mim]NTf_2_. It might be explained as follows: compared with methanol, ILs, especially those containing halogen anions, have shown high capacity for cellulose dissolution, which is conducive to the release of active substances from the cells of medicinal plants [23]. But at the same time, the viscosity of ILs also has a great influence on the extraction rate of FA in the solid samples. Therefore, [C_4_mim]NTf_2_ has the highest extraction yield of FA due to its lowest viscosity and the easy formation of homogeneous turbid IL–water solution during the extraction.

Due to its acceptable hydrophobicity and low viscosity, [C_4_mim]NTf_2_ can be dispersed completely into the aqueous solution during the extraction process and well separated in the centrifugation process. Since [C_4_mim]NTf_2_ exhibits the highest extraction ability for FA, it is selected as the extraction solvent in the following studies.

#### 3.1.2. Effect of IL Volume

**Figure 6:**
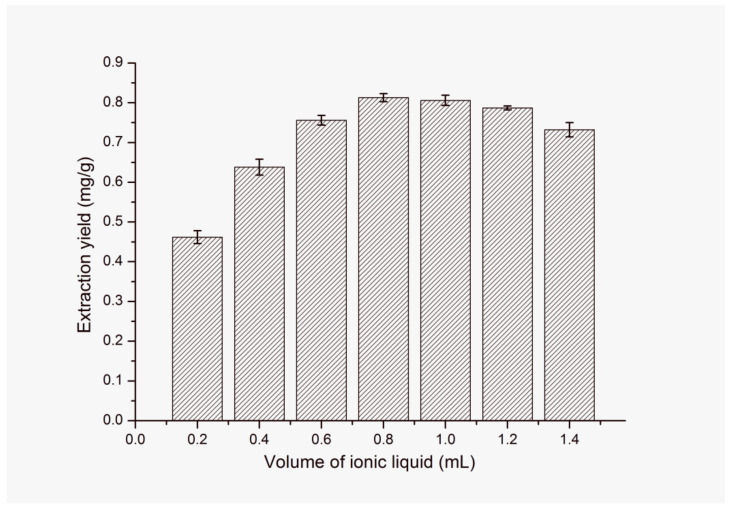
Effect of ionic liquid (IL) volume on extraction yield of FA. Sample amount, 50 mg; type of IL, [C_4_mim]NTf_2_; extraction temperature, 60 °C; solid–liquid ratio, 1:40 (g/mL); ultrasonic time, 9 min; centrifugal speed, 11,000 rpm; centrifugal time, 5 min.

The effect of different volume of [C_4_mim]NTf_2_ on the extraction ability was studied when the total volume of extractant ([C_4_mim]NTf_2_ and water) was maintained at 2 mL. The results shown in Figure 6 indicate that the volume of [C_4_mim]NTf_2_ is closely related to the extraction yield of FA. With the increase of volume of [C_4_mim]NTf_2_ from 0.2 mL to 1.4 mL, the extraction yield of target analyte increases gradually and reaches the maximum extraction yield at 0.8 mL of [C_4_mim]NTf_2_. Then, with the further increase of volume of [C_4_mim]NTf_2_ from 0.8 mL to 1.4 mL, the extraction yield of FA actually slightly decreases. This can be explained as follows: as the volume of [C_4_mim]NTf_2_ increases, more [C_4_mim]NTf_2_ is dispersed into water under the drive of temperature and ultrasound. As a consequence, the interactions among [C_4_mim]NTf_2_ and FA are improved, which leads to the increase of extraction yield. However, when the volume of [C_4_mim]NTf_2_ is more than 0.8 mL, excess of [C_4_mim]NTf_2_ might exceed the target analyte dispersed amount. At the same time, the viscosity of the extractant would increase, which is not beneficial to the mass transfer. Moreover, excessive [C_4_mim]NTf_2_ would be adsorbed on the solid sample, which could not only lead to poor infiltration of the solvent into the plant tissues but also make phase separation difficult during the centrifugation, resulting in the decrease of the extraction yield. Thus, 0.8 mL of [C_4_mim]NTf_2_ is finally selected for the following experiments.

#### 3.1.3. Effect of Solid–Liquid Ratio

**Figure 7:**
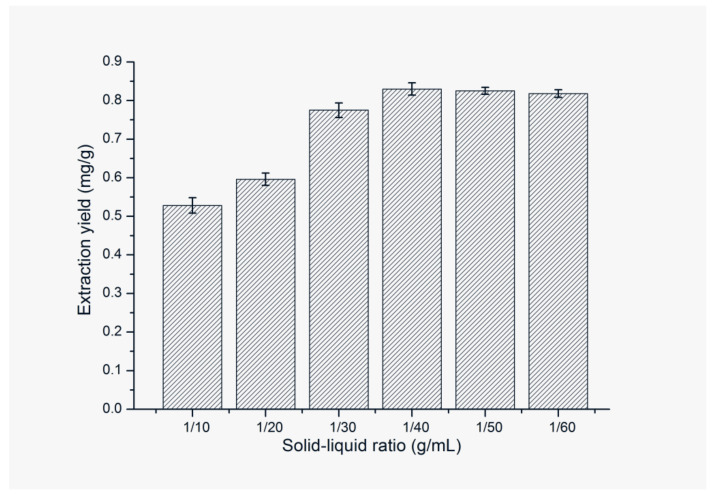
Effect of solid–liquid ratio on extraction yield of FA. Sample amount, 50 mg; type of IL, [C_4_mim]NTf_2_; extraction temperature, 60 °C; volume of IL, 0.8 mL; ultrasonic time, 9 min; centrifugal speed, 11,000 rpm; centrifugal time, 5 min.

Solid–liquid ratio is also an important factor affecting the extraction efficiency. Therefore, the effect of solid–liquid ratios on the extraction efficiency was investigated by using [C_4_mim][NTf_2_]-water (containing 40% (volume ratio) of [C_4_mim][NTf_2_]) as extractant. The results shown in Figure 7 illustrate that the extraction efficiency of FA increases with the increase of solid–liquid ratio from 1:10 (g/mL) to 1:40 (g/mL) and then keeps almost constant with further increasing the solid–liquid ratio. It means that when the solid–liquid ratio is 1:40 (g/mL), the target analytes in the sample are extracted completely by ILs. Therefore, 1:40 (g/mL) is chosen for the optimal solid–liquid ratio to avoid the waste of reagents.

#### 3.1.4. Effect of Extraction Temperature

**Figure 8:**
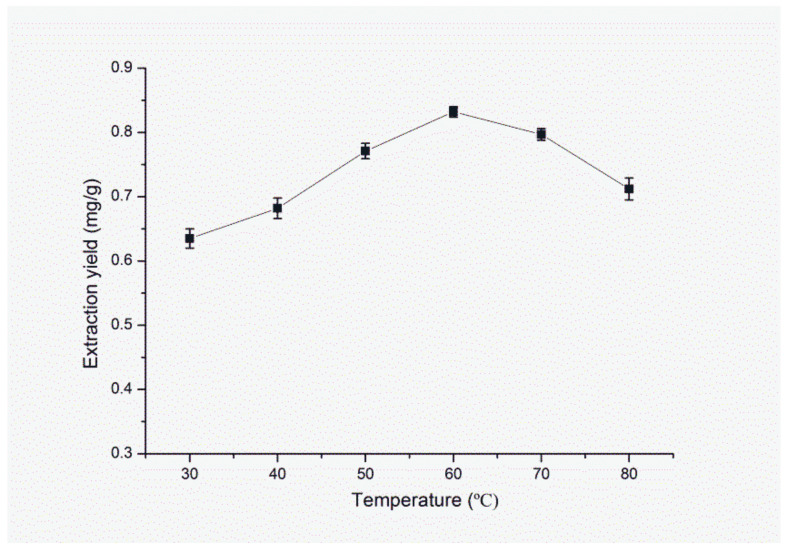
Effect of extraction temperature on extraction yield of FA. Sample amount, 50 mg; type of IL, [C_4_mim]NTf_2_; solid–liquid ratio, 1:40 (g/mL); volume of IL 0.8 mL; ultrasonic time, 9 min; centrifugal speed, 11,000 rpm; centrifugal time, 5 min.

In the proposed method, temperature is one of the important driving force for the complete dispersion of [C_4_mim][NTf_2_] into the water and affects the mass transfer. Thus, a series of experiments was designed to investigate the effect of temperature on the extraction efficiency and the results are shown in. It can be observed from Figure 8 that the extraction efficiency of FA increases with the increase of temperatures from 30 °C to 60 °C. When the temperature is in the range of 70 °C to 80 °C, the extraction efficiency of FA is reduced as compared with that at 60 °C. It might be due to the degradation of FA and the increasing solubility of the IL into water, which is negative to phase separation. Therefore, 60 °C is used as the optimum extraction temperature for the future studies.

#### 3.1.5. Effect of Ultrasound Time

**Figure 9:**
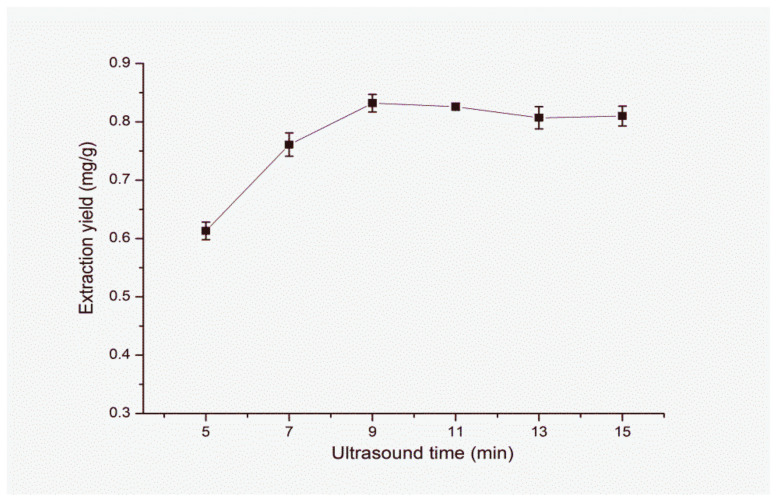
Effect of ultrasound time on extraction yield of FA. Sample amount, 50 mg; type of IL, [C_4_mim]NTf_2_; extraction temperature, 60 °C; volume of IL, 0.8 mL; solid–liquid ratio, 1:40 (g/mL); centrifugal speed, 11,000 rpm; centrifugal time, 5 min.

Ultrasound time is another leading factor influencing the extraction yield to certain extend. As shown in Figure 9the extraction yield of FA increases with the extension of ultrasound time from 5 to 9 min. When the ultrasound time continues to increase, the extraction efficiency of the FA remains almost unchanged. The rapid extraction may be ascribed to the synergistic effect of ultrasound and heating during the extraction process. Accordingly, the ultrasound time of 9 min is adopted in the following study.

#### 3.1.6. Effect of Centrifugal Speed and Time

**Figure 10:**
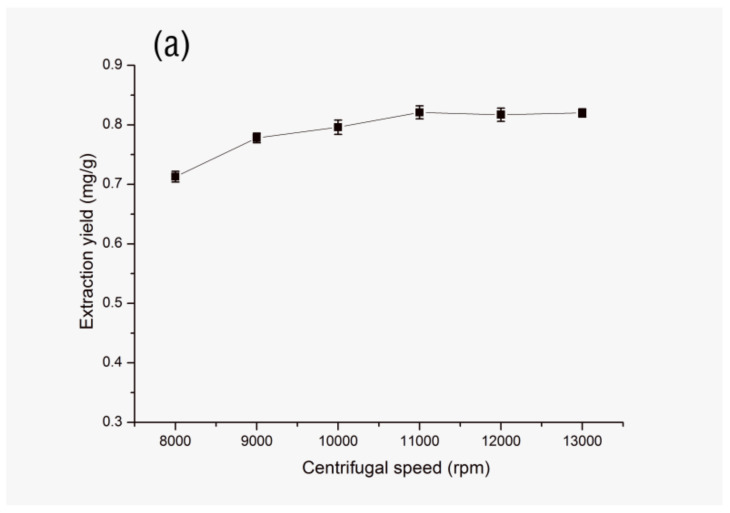

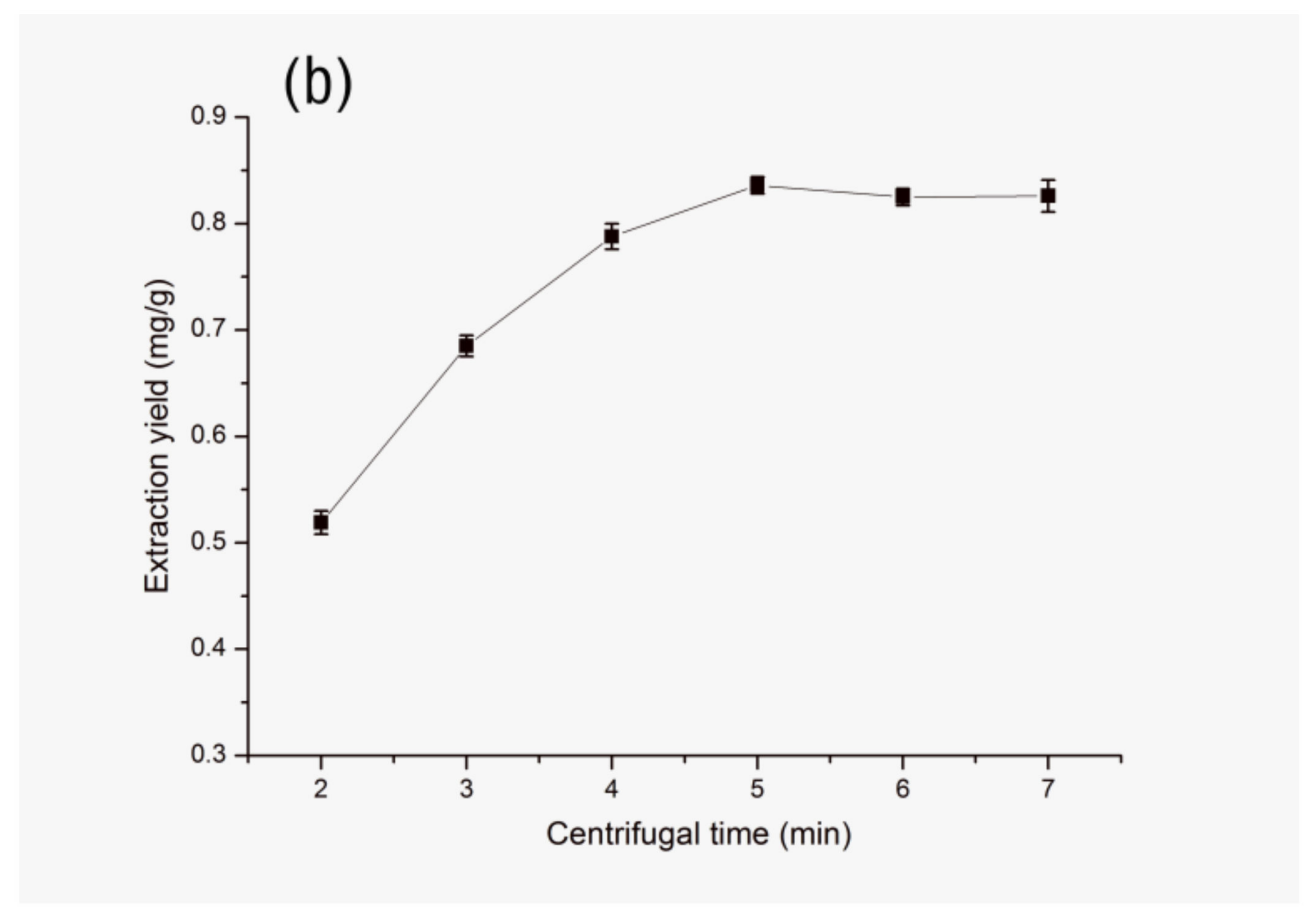
Effect of centrifugal speed (**a**) and time (**b**) on extraction yield of FA. Sample amount, 50 mg; type of IL, [C_4_mim]NTf_2_; extraction temperature, 60 °C; volume of IL, 0.8 mL; solid–liquid ratio, 1:40 (g/mL); ultrasonic time, 9 min.

In order to achieve better phase separation, centrifugation was performed at 0 °C. At the same time, the effect of centrifugal speed (8000, 9000, 10,000, 11,000, 12,000 and 13,000 rpm) and centrifugal time (2, 3, 4, 5, 6 and 7 min) on the extraction efficiency were investigated. As it can be seen in Figure 10 (a) and (b), the extraction efficiency of FA is the highest and complete phase separation can be achieved when the centrifugal speed is 11,000 rpm and the centrifugal time is 5 min.

### 3.2. Comparison of the Proposed TC-ILs-UHAE with the Conventional Methods

**Figure 11:**
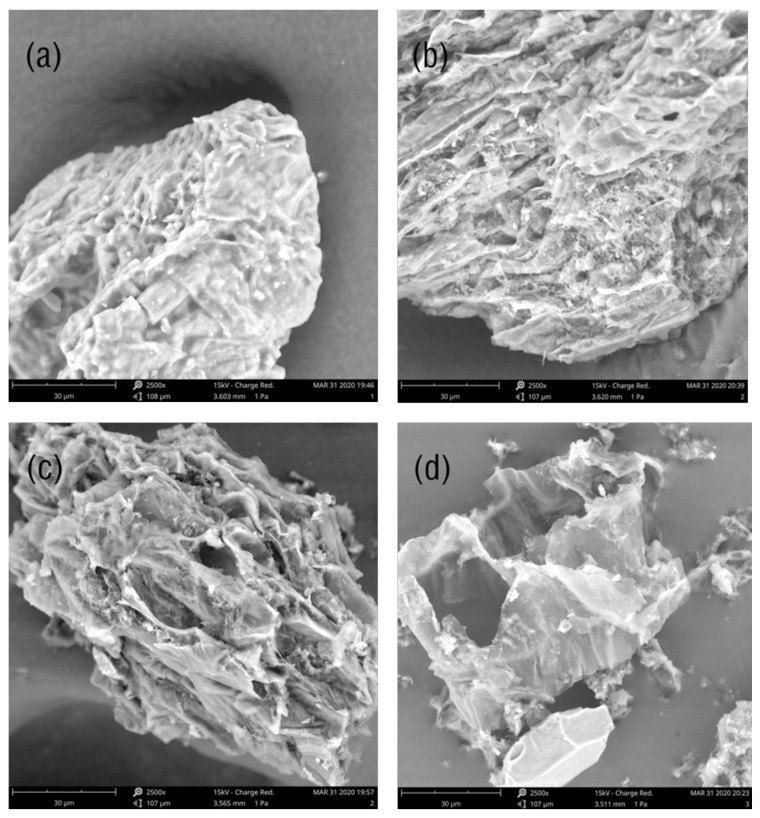
Scanning electron microscopy (SEM) images of *Angelica sinensis* sample powder (**a**) before extraction, (**b**) after heated reflux extraction (HRE), (**c**) after Pharmacopoeia method, (**d**) after TC-IL-UHAE.

To evaluate the extraction efficiency of different methods, the proposed TC-ILs-UHAE approach under optimal conditions was compared with the optimized conventional methods, including HRE and Pharmacopoeia methods. The results shown in Table 1 indicate that the proposed TC-ILs-UHAE method exhibits the highest extraction efficiency and the shortest extraction time among the three extraction methods. This could be attributed to the synergistic effect of heating and ultrasound in the proposed method. To verify this hypothesis, the SEM images of outer surface of the *Angelica sinensis* powders before and after different extraction procedures were captured and compared. The results are illustrated in Figure 11a–d.

The raw *Angelica sinensis* powder in Figure 11a displays a rough and complete surface with numerous protruding blunt horn-like lumps. Figure 11b,c are the images after extraction using HRE and Pharmacopoeia methods, respectively. It can be seen that the surface microstructure of the sample is obviously damaged under the influence of heating. Sample submitted to TC-ILs-UHAE method is damaged thoroughly and presents more fragmented structures and ruptured plant cell under the synergistic effect of heating and ultrasound (Figure 11d). High temperature increases the kinetic energy of molecules and is more conducive to the diffusion of FA. At the same time, ultrasound-treated cells have a more open, fragmented structure, which facilitates the access of the solution into the cellular compartments where the target analyte is located. Although the extraction yield of pharmacopoeia method is similar to that of our proposed method, the extraction time of pharmacopoeia method is longer and environment-hazardous methanol is used as the extraction solvent. Moreover, compared with HRE, much less extraction time and solvent are consumed, and more extraction yield is obtained when the present method is applied. Therefore, the proposed method is considered as a comparatively satisfactory technique.

### 3.3. Analytical Performance

Under the optimized conditions, some parameters such as linearity, limits of detection (LOD, *S*/*N* = 3), and reproducibility of the proposed method were investigated. The calibration curve was constructed by plotting the peak areas versus the concentration of FA and obtained by the analysis of six concentrations of the standard FA solutions in triplicate. The calibration equation was *y* = 38022.5 *x* + 1829.2 with a correlation coefficient (*r*) of 0.9995 in the concentration range of 0.25 to 25.00 mg/L. The limit of detection (LOD) based on signal-to-noise ratio of 3 (*S*/*N* = 3) is 9.6 µg/L, which indicated the developed method is of satisfactory sensitivity. The reproducibility was investigated by the determination of six replicated extracts of the sample 1 and the relative standard deviation (RSD, *n* = 6) value was 3.20%.

To test the accuracy of the proposed method, the spiked sample 1 with three levels were determined. The recovery could be calculated as follows:

(2) $$\mathrm{Recovery}\;(\%)=\frac{\mathrm{found}\;\mathrm{yield}\;(\mathrm{mg}/g)-\mathrm{original}\;\mathrm{yield}\;(\mathrm{mg}/g)}{\mathrm{spiked}\;\mathrm{concentration}\;(\mathrm{mg}/g)}\times 100$$

The results shown in Table 2 indicate that good recoveries ranging from 91.67% to 102.00% are obtained and the precision (expressed as RSD, *n* = 5) is lower than 3.87%.

### 3.4. Analysis of Real Samples

In order to verify the applicability of the proposed method, six batches of *A. sinensis* samples from different cultivated areas of China were analyzed under the optimal extraction conditions. Results in Table 3 indicate that the content of FA from Gansu province is obviously higher than that from Yunnan province, which suggests that the quality of *A. sinensis* is related to its cultivated regions.

## 4. Conclusions

In the present work, an efficient, environmentally friendly and easily operated TC-ILs-UHAE method using hydrophobic IL [C_4_mim][NTf_2_] as extraction solvent was developed. The influence of the IL anions and cations on the extraction yield was systematically studied. Among all the ILs used in this work, [C_4_mim]NTf_2_ exhibited the highest extraction efficiency due to its relatively higher hydrophobicity and lower viscosity and weaker steric hindrance effect. Compared with the traditional HRE and Pharmacopoeia methods, the present method has higher extraction efficiency and shorter extraction time due to the synergistic effect of simultaneous ultrasound and heating, which was confirmed by SEM images of the samples. Additionally, separation and enrichment could be incorporated into the centrifugation process without extra ice bath and additional salt, which is conducive to saving time and easy operation. Moreover, the present TC-ILs-UHAE method has good analytical performances and has been successfully applied to the analysis of real herbal samples from different cultivated regions. Since hydrophobic ILs were rarely directly used to extract solid samples, this proposed method provides an alternative and promising approach for analysis and separation of active components from other solid natural products.

## Figures and Tables

**Table 1:** Comparison of Temperature-Controlled Hydrophobic Ionic Liquids-Based Ultrasound/Heating-Assisted Extraction (TC-ILs-UHAE), Heated Reflux Extraction (HRE) and Pharmacopoeia Method.

Method	Extraction Yield (mg/g)	Sample Amount (mg)	Solvent	Volume of Solvent (mL)	Extraction Time (min)
TC-ILs-UHAE	0.83 ± 0.015 ^a^	50	[C_4_mim]NTf_2_	0.8	9
HRE	0.68 ± 0.011	200	[C_4_mim]NTf_2_	8	60
Pharmacopoeia	0.81 ± 0.012	200	Methanol	20	30

^a^ Mean ± standard deviation, *n* = 3; [C_4_mim]NTf_2_—1-butyl-3-methylimidazolium bis(trifluoromethanesulphonyl).

**Table 2:** Recoveries of Ferulic Acid (FA) in Spiked Sample 1.

	Original (mg/g)	Added (mg/g)	Recovery (%)	RSD (%, *n* = 5)
FA	0.83	1.00	102.00	2.65
		0.80	93.75	1.53
		0.60	91.67	3.87

**Table 3:** Analysis of Real Samples.

Sample Number	Cultivation Region	Extraction Yield (mg/g)	RSD (%, *n* = 5)
1	Gansu	0.83	3.15
2	Gansu	0.86	2.26
3	Gansu	0.82	2.87
4	Gansu	0.78	1.55
5	Yunnan	0.66	3.62
6	Yunnan	0.73	1.68
